# A Battery-Free, Data-Informed UV Dose Sensor Made of Laser-Induced Graphene and Bio-Derived Electrolytes †

**DOI:** 10.3390/mi17030302

**Published:** 2026-02-28

**Authors:** Mohammadreza Chimerad, Pouya Borjian, Faisal Bin Kashem, Swaminathan Rajaraman, Hyoung J. Cho

**Affiliations:** 1 Department of Mechanical & Aerospace Engineering, College of Engineering & Computer Science, University of Central Florida, Orlando, FL 32816, USA; mohammadreza.chimehrad@ucf.edu (M.C.); pouya.borjian@ucf.edu (P.B.); 2 Department of Electrical and Computer Engineering, NanoScience Technology Center, University of Central Florida, Orlando, FL 32816, USA; faisal@ucf.edu (F.B.K.); swaminathan.rajaraman@ucf.edu (S.R.); 3 Department of Materials Science and Engineering, Biomedical Engineering Program, and Burnett School of Biomedical Sciences, University of Central Florida, Orlando, FL 32816, USA

**Keywords:** laser-induced graphene, UV sensors, ionochromic cell, intelligent food packaging, sustainable materials, dose modeling, flexible sensors

## Abstract

This study presents a sustainable, battery-free UV (ultraviolet) dose sensor designed for intelligent food packaging applications. The device integrates laser-induced graphene (LIG) electrodes, a ZnO-CNT (carbon nanotube) UV-active composite, and a bio-derived ionochromic cell composed of blueberry anthocyanins and a NaCl electrolyte. This work advances the platform by introducing a quantitative and predictive dose–color mapping framework for cumulative UV detection under zero-bias operation. A controlled charge-injection protocol was employed to emulate UV-generated photocurrent, enabling systematic investigation of charge-transfer-driven ionochromic kinetics across five current levels (0.2–3 mA). HSB (hue–saturation–brightness)-based colorimetric analysis was performed to quantify the time-dependent chromatic evolution, and a numerical fitting model was developed to map charge accumulation to color shifts. Using this calibration, the color response at microampere-level photocurrents—corresponding to real zero-bias UV operation—can be predicted. The resulting model enables estimation of the cumulative time required for the ionochromic cell to transition from red to purple under realistic UV intensities. By combining self-powered sensing with predictive colorimetric modeling, this work significantly enhances the functionality of battery-free UV indicators, enabling quantitative dose measurement without external electronics for safer food-supply-chain monitoring.

## 1. Introduction

Ensuring food quality and safety throughout storage and distribution requires continuous monitoring of environmental conditions that directly affect product freshness [1,2,3]. Conventional packaging materials are passive systems that cannot provide feedback about exposure to light, temperature changes, or biochemical degradation [4,5,6]. In response, the field of intelligent packaging has explored colorimetric indicators—simple, low-cost visual tools that can reveal spoilage events or storage history [7]. Among these, indicators based on natural pigments, such as anthocyanins, have gained significant attention due to their biodegradability, pH sensitivity, and strong visual contrast, making them suitable for real-time freshness monitoring. In particular, blueberry-derived anthocyanins offer a high pigment concentration and a pronounced, well-defined pH-dependent chromatic transition across the acidic-to-alkaline range, while maintaining food safety and compatibility with sustainable packaging systems [8,9,10]. Existing systems, however, primarily respond to pH shifts, gas release, or temperature variations. At the same time, the ability to track cumulative UV exposure remains largely unaddressed, despite UV radiation’s role in accelerating oxidation, nutrient loss, and the degradation of packaging materials [11,12].

Time–temperature indicators (TTIs) provide a helpful analogy because they quantify a product’s cumulative thermal history through kinetically driven color changes that reflect its shelf life [13]. However, an equivalent indicator for UV dose—one that visually records the total amount of absorbed optical energy—does not yet exist in a compact, low-cost, and sustainable packaging-compatible form. Current colorimetric systems offer instantaneous responses but lack a mechanism to integrate UV exposure over time [11]. This limitation underscores the need for a device that converts UV energy into a stable, quantifiable visual signal.

Existing UV indicators and electronic UV dosimeters primarily provide instantaneous or intensity-dependent responses and typically rely on external power sources or electronic readout systems, which limits their suitability for long-term, low-cost integration into sustainable packaging. In contrast, the platform presented in this work enables cumulative UV dose memory by directly converting absorbed optical energy into a persistent ionochromic color state under battery-free, zero-bias operation. By integrating laser-induced graphene electrodes, a ZnO-based photoactive layer, and a bio-derived ionochromic cell, this approach uniquely combines self-powered operation with chromatic dose storage and predictive dose–color modeling, distinguishing it from existing UV sensing and indication technologies.

Our previous work [14] introduced a miniaturized, battery-free UV sensor–actuator that accomplishes this by coupling a ZnO (zinc oxide)–CNT (Carbon nanotubes)/LIG (Laser-induced graphene) photodetector with an anthocyanin-based ionochromic cell, utilizing eco-friendly, sustainable materials. While laser irradiation can enable a wide range of laser–matter interaction mechanisms depending on the processing regime, in this work it is employed specifically for localized photothermal graphitization of polymer substrates to fabricate laser-induced graphene electrodes. Under UV illumination, the device generates a small, zero-bias photocurrent that drives electrochemical reactions within the ionochromic reservoir, producing a visible red-to-purple color shift proportional to the accumulated charge—and therefore to the cumulative UV dose. This unique mechanism enables the device to operate without external power, making it fundamentally different from conventional electronic dosimeters and suitable for integration into sustainable smart packaging.

The present work presents a controlled charge-injection calibration method and a custom ionochromic fixture that isolates the intrinsic color-change kinetics from UV variability. By applying defined currents and extracting HSB evolution over time, we construct a quantitative charge–color relationship and use numerical fitting to predict the device response at microampere-level currents typical of real UV operation. This expanded analysis provides a mechanistic understanding of the ionochromic transition and establishes a predictive framework for UV-dose estimation, advancing the device toward practical intelligent-packaging applications.

## 2. Materials and Methods

### 2.1. Materials

Kapton polyimide films (127 μm thickness, DuPont) were purchased from CS Hyde (Lake Villa, IL, USA). Zinc oxide (ZnO) nanopowder (≤100 nm), terpineol, sodium chloride (ACS grade), and polypropylene (PP) films were obtained from Fisher Scientific (Waltham, MA, USA). Multi-walled carbon nanotubes (CNTs, 20–40 nm diameter) and ethyl cellulose (EC) were sourced from TCI Chemicals (Tokyo, Japan). Tin (II) chloride (SnCl_2_), hydrochloric acid (HCl), and polyethylene glycol (PEG) were purchased from Sigma-Aldrich (St. Louis, MO, USA). Commercial dried seaweed sheets were obtained from a local market. Fresh blueberries were used as received. Unless otherwise stated, all materials and chemicals were used without further purification.

### 2.2. Sensor Fabrication

**Figure 1:**
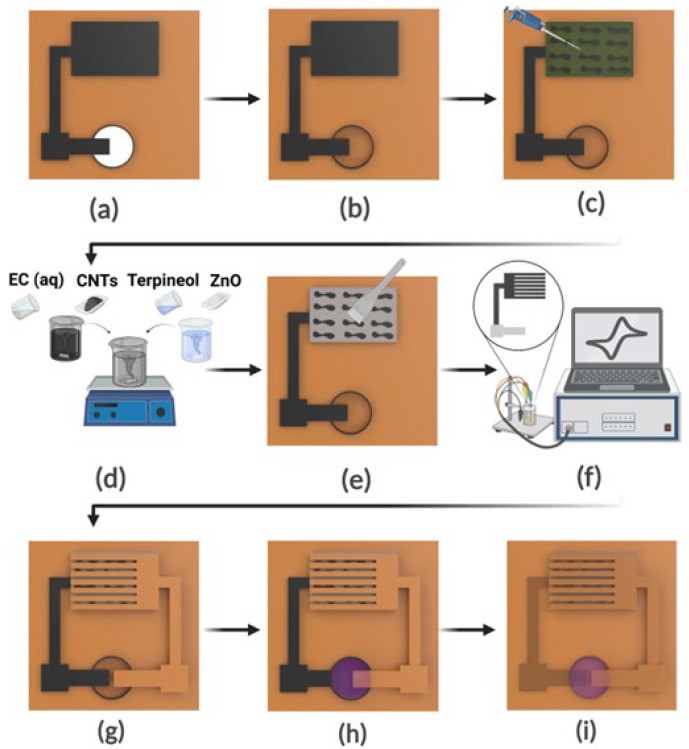
Fabrication steps for the UV dose sensor (not to scale): (**a**) patterning of the first LIG electrode, (**b**) bonding a Kapton spacer layer beneath the electrode to define the ionochromic reservoir, (**c**) laminating a NaCl-hydrated seaweed sheet onto the assembly, (**d**) preparing the ZnO–CNTs composite solution, (**e**) depositing the ZnO–CNTs layer onto the seaweed substrate, (**f**) selectively electroplating the terminal region of the second LIG electrode with Sn, (**g**) flipping and aligning the second electrode onto the spacer to complete the cavity structure, (**h**) injecting the blueberry extract/NaCl electrolyte into the reservoir, and (**i**) sealing the device with a transparent polypropylene(PP) film.

As shown in Figure 1, interdigitated LIG electrodes were first patterned on Kapton polyimide films using CO_2_ laser irradiation (4.5 W, 200 mm/s, 500 PPI), which carbonizes the polymer surface to form a porous, conductive graphene network consistent with our earlier methodology. These laser parameters were optimized to achieve consistent photothermal graphitization and to minimize the sheet resistance of the resulting laser-induced graphene electrodes, ensuring reliable electrical performance across devices (Table 1).

The listed parameters were selected to ensure reproducible graphitization and minimized sheet resistance of the laser-induced graphene electrodes.

To prepare the UV-sensitive layer, CNTs were dispersed in a terpineol/ethyl cellulose mixture via ultrasonication, and ZnO nanopowder was subsequently added at a 10:1 ZnO: CNTs mass ratio to form a uniform composite. This aqueous paste was spread onto a NaCl-hydrated seaweed sheet placed on the lower LIG electrode and allowed to dry under ambient conditions, thereby forming the ZnO–CNTs photoresponsive coating. The upper LIG electrode was then selectively metalized by electrodepositing tin from an aqueous bath containing 1 M SnCl_2_, 0.1 M HCl, and 0.01 M PEG, at a constant current of 10 mA and an electrode spacing of 8 mm.

A laser-cut Kapton spacer defining the ionochromic cavity was laminated onto the lower electrode, after which the Sn-coated upper electrode was aligned and bonded to form the enclosed cell configuration. A mixture of blueberry extract and a NaCl solution (4:1 *v*/*v*), rich in anthocyanin pigments, was injected into the cavity as the ionochromic electrolyte. The assembled device was finally sealed with a transparent PP (polypropylene) adhesive film to prevent evaporation and allow direct visual observation of the colorimetric response. The resulting structure integrates the LIG electrodes, the ZnO–CNTs UV-sensing composite, the NaCl-infused seaweed ionic layer, the anthocyanin reservoir, and the Sn working electrode into a fully integrated sensing module.

It is important to note that although Kapton itself is not transparent to UV, the device architecture is designed so that UV illumination interacts primarily with the exposed interdigitated gaps between the LIG electrodes rather than propagating through the Kapton substrate. These gaps are intentionally enriched with the ZnO–CNTs photoactive composite, enabling incident UV radiation to excite the semiconductor layer and directly generate photocurrent.

### 2.3. Experimental Methods

#### 2.3.1. UV Illumination and Photodetector Measurement Setup

The UV response of the ZnO–CNTs/LIG photodetector was evaluated using a 365 nm UV irradiation lamp positioned at a fixed distance to ensure uniform illumination across the device surface. The irradiance at the measurement plane was maintained at 2 mW·cm^−2^ for all experiments. Photocurrent measurements were performed under zero-bias conditions using a Keithley 2401 Source Meter (Keithley Instruments, Solon, OH, USA), operated in current-measurement mode with variable applied voltages when required. Time-dependent photocurrent responses were recorded by periodically switching the UV source on and off while continuously monitoring the output current. All measurements were conducted at room temperature under ambient laboratory conditions.

#### 2.3.2. Ionochromic Calibration Fixture

**Figure 2:**
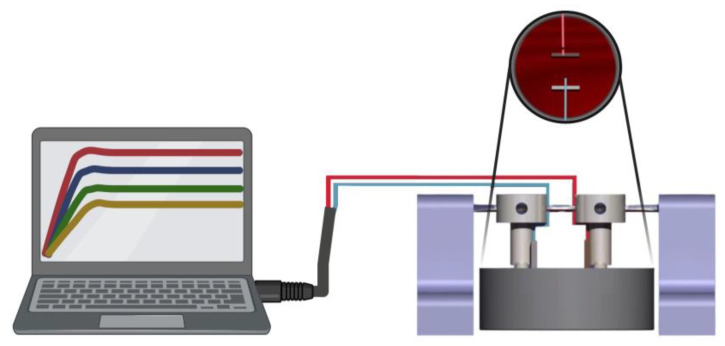
Schematic illustration of the custom-built calibration fixture used for controlled charge-injection and optical monitoring of the ionochromic response.

To obtain reproducible and isolated ionochromic responses independent of UV intensity, a custom-designed calibration fixture was developed to standardize the measurement environment. The fixture was first modeled using computer-aided design (CAD) software, SolidWorks (Dassault Systèmes, version 2024). and subsequently fabricated with a Formlabs 3D printer using clear photopolymer resin, enabling tight dimensional tolerances and optical accessibility during imaging. As demonstrated in Figure 2, during galvanostatic charge-injection experiments, the fixture securely holds the plated electrodes in a fixed geometry. It maintains a well-defined electrode spacing while electrically mimicking the charge-transfer conditions within the sealed sensor cavity. The structure minimizes ambient-light interference and controls the optical path for image acquisition by providing a stable, enclosed, and reproducible measurement environment.

#### 2.3.3. Controlled Charge-Injection Protocol

The ionochromic behavior was calibrated by electrically simulating the charge accumulation that occurs during UV exposure. Controlled charge injection was performed using a PalmSens potentiostat operated in galvanostatic (constant-current) mode. This protocol was intentionally designed to replicate the photocurrent generated by the ZnO–CNT/LIG photodetector under UV illumination and subsequently transfer it to the ionochromic cell during zero-bias operation. By directly controlling the injected current and duration, the accumulated charge delivered to the ionochromic electrolyte could be precisely defined, enabling systematic isolation of the ionochromic response from variations in UV intensity and photodetector dynamics.

#### 2.3.4. Image Acquisition and HSB Color Analysis

Colorimetric changes in the ionochromic reservoir were recorded at fixed time intervals using a camera mounted on a stable platform to maintain constant imaging geometry throughout the experiment. All images were captured under uniform illumination and identical exposure settings to eliminate variations caused by ambient lighting. A predefined Region of Interest (ROI) within the ionochromic cell was selected to ensure consistent color extraction across all time points.

HSB (hue–saturation–brightness) values were computed programmatically using Python (version 3.13) with the OpenCV library (version 4.12.0), following standard HSB syntax to extract the mean color intensity within the ROI. These values were then processed to quantify the temporal evolution of the ionochromic response at each applied current level. The resulting HSB trajectories served as the basis for subsequent numerical modeling, enabling prediction of low-current color behavior under realistic zero-bias UV operation. Specifically, mean HSB values were extracted at each time point and plotted as a function of time to generate time-resolved color trajectories for each applied current level.

## 3. Working Principle

**Figure 3:**
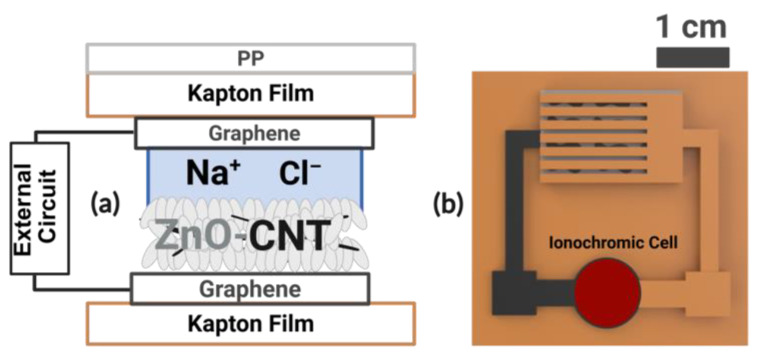
Working principle of the UV dose sensor (not to scale): (**a**) a side view showing UV-induced charge generation in the ZnO-CNT/LIG photodetector; (**b**) a top view showing a connection to an ionochromic cell, which produces a visible color change.

The operation of the UV dose sensor relies on coupling a zero-bias ZnO–CNTs/LIG photodetector to an anthocyanin-based ionochromic cell, enabling both instantaneous and cumulative UV monitoring. As illustrated in Figure 3 and described in our previous work [14], the ZnO–CNTs composite serves as the UV-responsive semiconductor, whereas the NaCl solution provides ionic stabilization and supports continuous charge transport.

Upon illumination with 365 nm UV light, photons with energy exceeding the ZnO bandgap (3.37 eV) excite electrons from the valence band to the conduction band within the ZnO nanoparticles. The embedded CNTs network facilitates rapid charge separation and transport, enabling efficient collection of photogenerated electrons and holes. This enhancement of current transport pathways is essential for generating the required potential and sustained charge flow to drive ion separation and electrochemical reactions within the coupled ionochromic cell under zero-bias operation. The presence of NaCl electrolyte improves interfacial charge mobility, enabling a measurable photocurrent to form even under zero external bias. The magnitude and duration of this photocurrent scale with the incident UV intensity and exposure time, enabling direct transduction of optical energy into electrical charge.

This photocurrent drives an electrochemical ionochromic reaction within the encapsulated electrolyte reservoir. The Sn-coated terminal on the upper LIG electrode functions as the anode of the ionochromic cell. Under UV-induced current flow, Sn undergoes oxidation according to:

(1) $$Sn\to {Sn}^{2+}+2e^{-},$$

While electrons delivered to the LIG cathode participate in the reduction in water:

(2) $$2H_{2}O\;+\;2e^{-}\to H_{2}+2{OH}^{-},$$

These half-reactions increase the local concentration of hydroxide ions in the blueberry–NaCl electrolyte, thereby shifting its pH. Anthocyanins, naturally occurring pigments extracted from blueberries, exhibit well-defined pH-dependent structural transitions, producing a visible color change from red to purple as the environment becomes more alkaline. Because the extent of this color transition is directly proportional to the total charge passed through the electrolyte, the system inherently records the integrated UV dose.

As a result, the device operates simultaneously as a real-time photodetector and a passive colorimetric memory element. The photodetector converts UV energy into electrical charge, while the ionochromic cell stores that charge as a persistent color shift. Once the charge-induced pH change occurs, the color state remains stable, providing a visual log of cumulative UV exposure.

## 4. Results and Discussion

### 4.1. Surface Morphology

**Figure 4:**
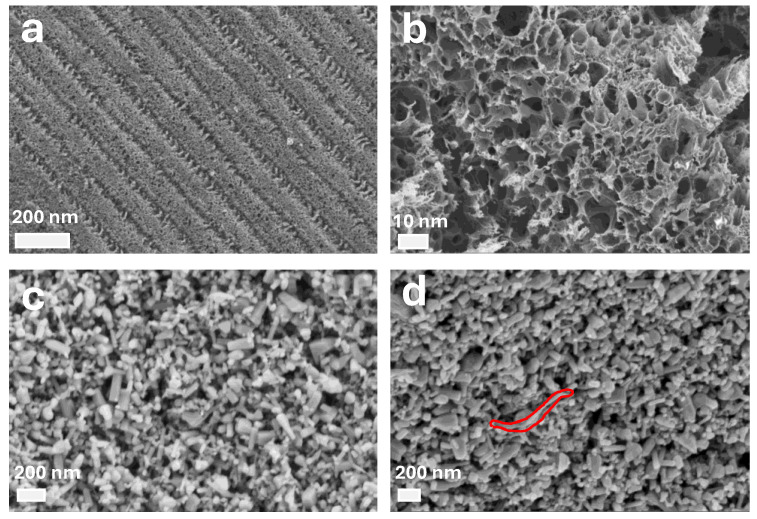
SEM images: (**a**,**b**) laser-induced graphene at two magnifications, and (**c**,**d**) the ZnO–CNT composite layer showing ZnO particles integrated with CNTs features highlighted by the red marking in (**d**).

Scanning electron microscopy (SEM) imaging was performed using a Zeiss NVision 40 system, operated at an accelerating voltage (EHT) of 4 kV and a working distance (WD) of 7.8 mm. SEM analysis revealed the surface morphology of the LIG electrodes and the ZnO-based photoactive layers. Figure 4a,b present SEM images of the LIG film at different magnifications. The low-magnification image shows the characteristic periodic laser-scribed ripple pattern, indicating uniform graphitization along the laser-scan direction within the CNTs composite layer. The pristine ZnO film exhibits densely packed nanocrystals with irregular geometries, forming a granular structure. In contrast, the ZnO–CNTs composite image reveals a hybrid morphology in which elongated CNTs bundles (highlighted) are embedded within the ZnO matrix.

### 4.2. Sensor Configuration

**Figure 5:**
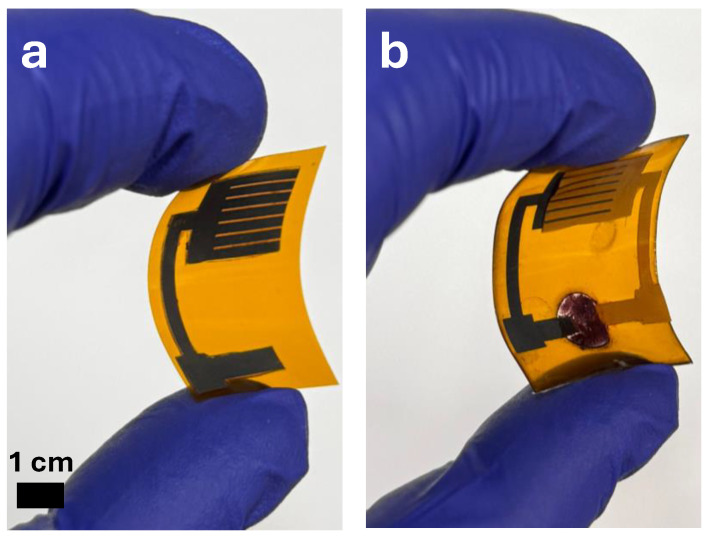
Fabricated device: (**a**) single patterned LIG electrode and (**b**) fully assembled UV sensor incorporating the ionochromic cell.

The UV dose sensor is presented in Figure 5. As shown in Figure 5a, the laser-patterned interdigitated LIG electrodes are cleanly defined on the flexible Kapton substrate, forming the conductive framework for UV detection. The patterned electrode geometry provides a large active surface area while preserving excellent mechanical flexibility. Figure 5b shows the fully assembled device, in which the LIG photodetector is coupled to the ionochromic reservoir, forming an integrated UV dose-sensing platform. The completed structure incorporates the ZnO–CNTs photoactive film, the NaCl-hydrated seaweed ionic layer, and the Sn-plated terminal that drives the charge-dependent color transition.

### 4.3. Optical Response and UV Dose Characteristics

**Figure 6:**
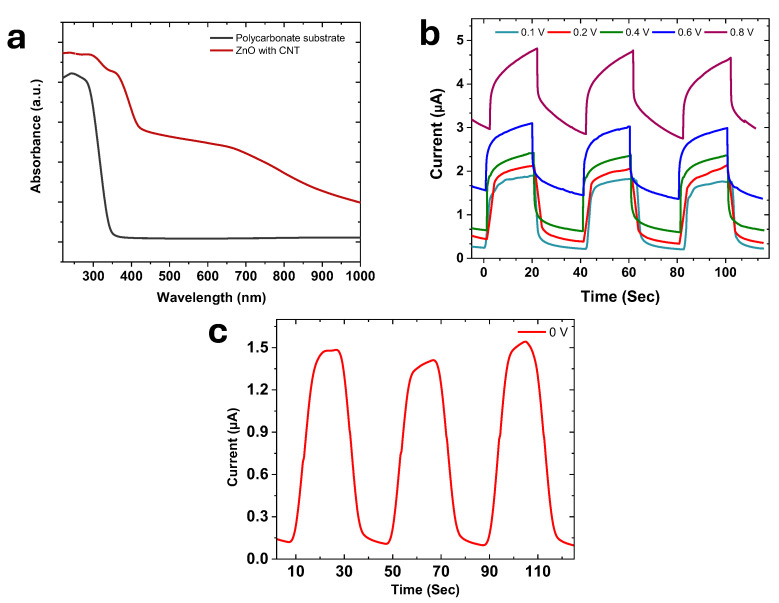
Optical and photoelectrical response of the UV dose sensor (**a**) UV-vis absorption spectra, (**b**) Time-dependent photoresponse under cyclic 365-nm UV irradiation at 0.1–0.8 V, and (**c**) 0 V bias conditions.

The optical and photoelectrical response of the UV dose sensor, including material-level absorption behavior and time-dependent photocurrent generation under UV illumination, is shown in Figure 6.

Figure 6a shows the UV–Vis absorption spectra of laser-induced graphene (LIG), LIG coated with ZnO, and the ZnO–CNTs composite. In the fabricated device, the ZnO-based sensing layer is selectively deposited within the gaps of the interdigitated LIG electrodes, which remain optically accessible after device sealing. As a result, UV light reaches the active ZnO–CNTs region through these exposed electrode gaps, rather than through the graphene itself. Accordingly, UV–Vis measurements were performed on the ZnO-containing layer, supported on the same transparent polypropylene film used for device encapsulation, to accurately reflect the optical path in the final sensor configuration. The ZnO–CNTs composite further enhances UV absorption intensity, which can be correlated to increased light scattering and improved electronic coupling provided by the CNT network.

Figure 6b shows the time-dependent photoresponse of the sensor under cyclic 365-nm illumination at different applied voltages. As the forward bias increases (0.1–0.8 V), the photocurrent magnitude rises correspondingly, demonstrating improved separation and transport of photogenerated carriers within the ZnO–CNTs composite. Each illumination cycle produces sharp, repeatable ON/OFF transitions, confirming the device’s reliability under externally applied bias.

The self-powered behavior of the sensor is highlighted in Figure 6c, which displays the photocurrent response under intermittent UV illumination at zero bias. In the absence of external voltage, the device still generates a consistent, reproducible photocurrent, driven solely by the built-in field at the interface among the ZnO nanoparticles, the CNTs network, and the LIG electrode. The stable photocurrent amplitude and rapid recovery indicate excellent carrier mobility and efficient charge extraction in the composite layer. Notably, the zero-bias operation produced a sensitivity of ~1000%, calculated using:

(3) $$S\left(\%\right)=100\times \frac{I_{UV}-I_{Dark}}{I_{Dark}},$$

It is important to note that the device’s photoresponse is intrinsically governed by the wide bandgap of ZnO (3.37 eV), which limits effective photoexcitation to the ultraviolet region. Illumination with longer-wavelength visible light does not generate a measurable photocurrent, confirming that the observed electrical and ionochromic responses are dominated by UV excitation.

### 4.4. Ionochromic Color Evolution Under Controlled Charge-Injection

**Figure 7:**
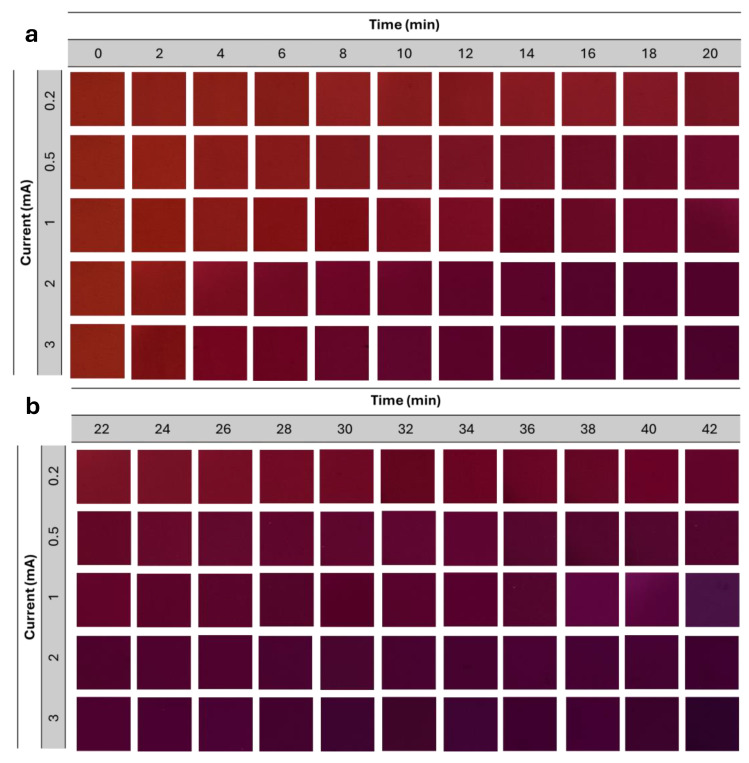
Time-dependent ionochromic color evolution under controlled charge injection at different current levels: (**a**) color transition recorded over the initial 0–20 min interval, and (**b**) extended color evolution from 22–42 min.

Figure 7 shows the time-dependent color evolution of the ionochromic cell under controlled charge injection at various current levels. At lower injection currents, the color transition proceeds gradually from red to reddish-purple, indicating a slower rate of proton consumption and a more gradual pH shift within the anthocyanin-based electrolyte. In contrast, increasing the applied current significantly accelerates the chromatic response, leading to deeper purple coloration over substantially shorter durations. This behavior is attributed to the higher charge flux delivered to the Sn electrode, which enhances the oxidation reaction and increases the local alkalinity within the ionochromic reservoir.

Each column in Figure 7a,b corresponds to a successive time interval, providing a clear visualization of the ionochromic reaction kinetics. The consistent, monotonic progression of color intensity across all current levels demonstrates the high controllability and reproducibility of the chromatic response. Notably, higher currents result in rapid color saturation, whereas lower currents produce smoother and more gradual transitions. This well-defined current-dependent behavior forms the basis for establishing a quantitative relationship between injected charge and color output, which is subsequently employed to develop the dose–color calibration and predictive modeling for zero-bias UV exposure.

**Figure 8:**
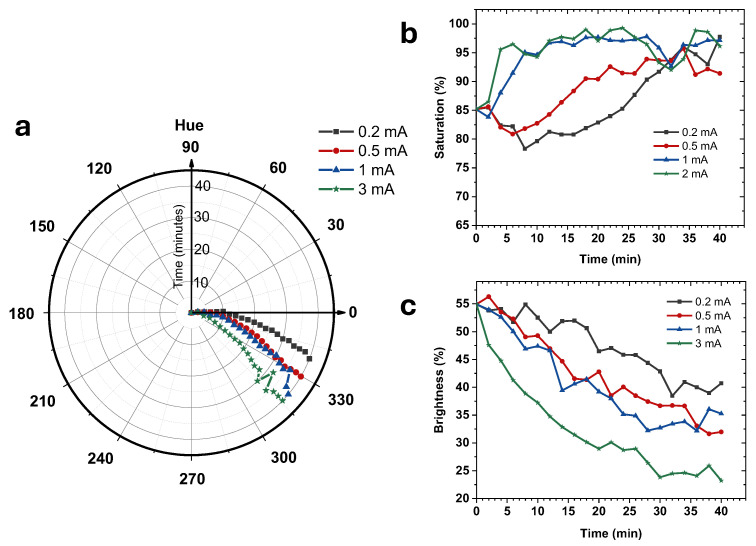
Quantitative HSB-based analysis of ionochromic color evolution under controlled charge injection. (**a**) Time-dependent variation in Hue, (**b**) Saturation, and (**c**) Brightness.

To quantitatively characterize this behavior, the captured images were converted into the HSB color space, and the temporal evolution of hue, saturation, and brightness was extracted, as shown in Figure 8. For each time point, hue, saturation, and brightness values were obtained by averaging the HSB values of all pixels within a fixed region of interest (ROI), producing time-resolved HSB trajectories.

The hue component exhibits a clear, systematic rotation over time, corresponding to the red-to-purple transition of the anthocyanin electrolyte (Figure 8a). Higher current levels induce faster hue shifts, indicating accelerated ionochromic kinetics under increased charge flux. In contrast, saturation increases during the color transition, reflecting enhanced chromatic contrast, while brightness decreases due to increased optical density as the electrolyte darkens (Figure 8b,c).

This behavior originates from the pH-dependent structural changes of anthocyanins. As the local alkalinity increases during charge injection, protons are progressively consumed, causing the anthocyanin molecules to change their chemical form. This transformation shifts the electrolyte color from red to purple by altering its light absorption characteristics. The simultaneous increase in color saturation and decrease in brightness arise from stronger light absorption as the colored species become more concentrated.

### 4.5. Dose-Color Calibration and Predictive Modeling

To develop a quantitative dose–color calibration model, HSB values extracted from controlled charge-injection experiments at 0.2, 0.5, 1, and 3 mA were used as training data for a linear regression model. These current levels span the operational range of the ionochromic cell and enable systematic mapping between accumulated charge and hue evolution. Importantly, the dataset obtained at 2 mA was intentionally excluded from the model construction and reserved exclusively for validation, ensuring that the proposed framework captures the underlying charge-dependent ionochromic kinetics rather than overfitting to specific experimental conditions.

**Figure 9:**
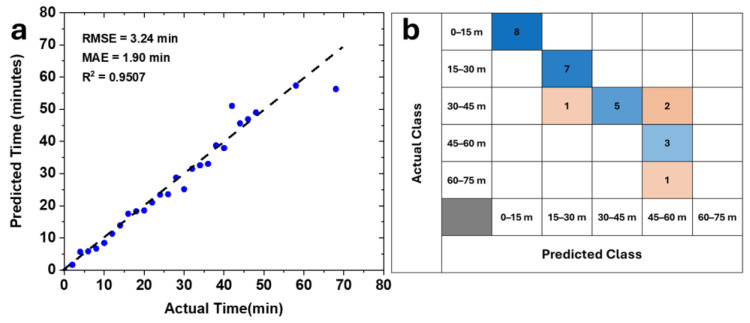
Dose-color calibration and validation of the predictive model, (**a**) Comparison of experimentally measured and model-predicted color-transition times, and (**b**) Confusion matrix illustration of time-binned prediction accuracy for 2 mA.

Model predictions were evaluated by comparing the predicted color-transition times with experimentally measured HSB values for the 2 mA dataset. As shown in Figure 9a, strong agreement is observed between predicted and actual times across the whole duration range. Quantitatively, the model achieves a root mean square error (RMSE) of 3.24 min and a mean absolute error (MAE) of 1.90 min, indicating high temporal prediction accuracy. The coefficient of determination (R^2^ = 0.9507) further confirms that the model captures the dominant trends governing ionochromic color evolution.

To assess practical usability, predicted times were additionally classified into 15-min temporal bins, yielding an overall prediction accuracy of 85.19%, as summarized by the confusion matrix in Figure 9b. Misclassifications are primarily confined to adjacent time bins, suggesting minimal error propagation and reinforcing the predictive framework’s robustness.

### 4.6. Prediction Dose-Color Change with Time Variation for Zero-Bias UV Operation

**Figure 10:**
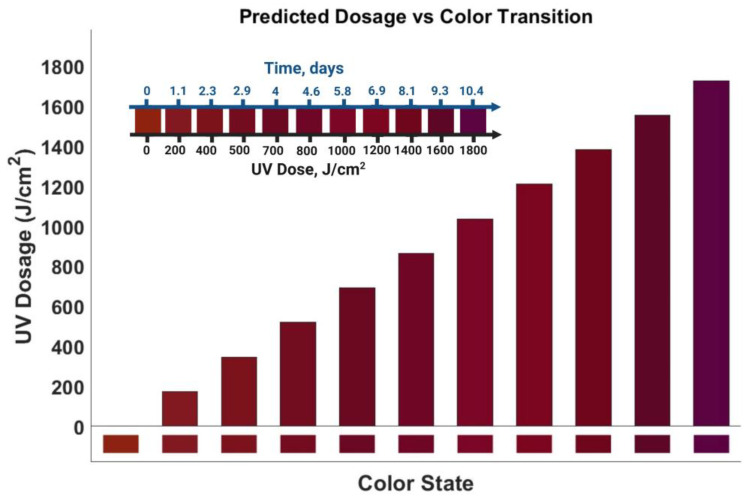
UV dose calibration chart based on the correlation between UV dose and color transition under zero-bias conditions. The ionochromic state evolves monotonically with accumulated UV dose at 0 V bias, while the secondary axis converts dose to exposure time at 2 $mW/{cm}^{2}$, demonstrating multi-day, self-powered dose integration.

Figure 10 presents the dose–color calibration map of the ionochromic UV dose sensor under zero-bias operation, establishing a quantitative relationship between the observed color state and the accumulated UV dose. The calibrated color evolution exhibits a gradual, monotonic transition from the baseline red state to progressively darker purple hues as the accumulated UV dose increases, confirming that the ionochromic response is governed by cumulative exposure rather than instantaneous intensity.

The calibration map indicates the onset of detectable color deviation from the initial state at an accumulated UV dose of approximately 200 J·cm^−2^, followed by a continuous, well-resolved chromatic progression up to 1800 J·cm^−2^. This monotonic behavior provides a robust basis for quantitative dose interpretation, ensuring that each color state corresponds uniquely to a specific range of accumulated UV exposure.

To facilitate practical interpretation, a secondary horizontal axis converts the accumulated UV dose to equivalent exposure time, assuming a constant irradiance of 2 mW·cm^−2^. Under these conditions, the calibrated color transition spans approximately 0–10.4 days of continuous UV exposure, demonstrating the device’s suitability for long-term, self-powered UV dose monitoring. Importantly, this dose–color calibration enables direct estimation of cumulative UV exposure from the visual color state under zero bias conditions.

## 5. Conclusions

This work presents a flexible, battery-free UV dose sensor that converts cumulative UV exposure into a persistent, visually interpretable color change. By integrating laser-induced graphene electrodes, a ZnO–CNT photoactive layer, and a bio-derived anthocyanin-based ionochromic cell, the device operates under zero-bias conditions and directly transduces optical energy into stored chromatic information.

The device architecture enables UV excitation through exposed interdigitated regions, while the ionochromic reservoir functions as a passive memory element that records accumulated dose without external power or electronics.

A controlled charge-injection strategy was introduced to electrically emulate UV-generated photocurrents and decouple ionochromic kinetics from illumination variability. Using HSB-based colorimetric analysis, a quantitative dose–color calibration model was established and independently validated, demonstrating strong predictive capability with low temporal error and high classification accuracy. This data-informed framework enables direct estimation of cumulative UV exposure from the observed color state under realistic zero-bias operation.

Overall, the presented approach establishes a scalable and sustainable pathway for quantitative, self-powered UV-dose monitoring and highlights the potential of bio-derived ionochromic systems for intelligent food packaging and other battery-free sensing applications.

One limitation of the present approach is that the sensor relies on an aqueous electrolyte, whose performance may be influenced by environmental factors such as temperature and humidity. Future work will focus on improving electrolyte stability and implementing appropriate strategies to mitigate environmental interferences and enhance long-term reliability in practical applications.

## Figures and Tables

**Table 1:** CO_2_ laser processing parameters used for LIG fabrication.

Parameter	Value
Laser type	CO_2_ laser
Laser power	4.5 W
Scand speed	200 mms^−1^
Pulses per inch (PPI)	500
Spot diameter	~100 µm
Pulse mode	Pulsed
Pulse repetition	System-defined
Substrate	Kapton polyimide
Processing atmosphere	Ambient air

## Data Availability

The data supporting this study’s findings are available from the corresponding authors upon reasonable request. The data are not publicly available due to ongoing research and planned future publications.

## Abbreviations

The following abbreviations are used in this manuscript:

UV	Ultraviolet
CNTs	Multi-Walled Carbon nanotubes
LIG	Laser-induced graphene
HSB	Hue–saturation–brightness
TTIs	Time-temperature indicators
ZnO	Zinc oxide
PP	Polypropylene
EC	Ethyl Cellulose
PEG	Polyethylene glycol
CAD	Computer-aided design
SEM	Scanning Electron Microscopy
RMSE	Root mean square error
MAE	Mean absolute error
